# Innovative Distance Learning Tool for Morphological Identification of Chigger Mites (Actinotrichida) as Vectors of Scrub Typhus: A Pilot Study

**DOI:** 10.3390/tropicalmed5020055

**Published:** 2020-04-05

**Authors:** Rawadee Kumlert, Aulia Rahmi Pawestri, Piyada Linsuwanon, Serge Morand

**Affiliations:** 1 The Office of Disease Prevention and Control 12, Songkhla Province (ODPC12), Department of Disease Control, Ministry of Public Health, Songkhla 90000, Thailand; rawadee.k@ddc.mail.go.th; 2 Faculty of Tropical Medicine, Mahidol University (FTM, MU), Bangkok 10400, Thailand; aulia_rp@ub.ac.id; 3 Faculty of Medicine, Universitas Brawijaya, Malang 65145, Indonesia; 4 United States Medical Directorate-Armed Forces Research Institute of Medical Sciences (US-AFRIMS), Bangkok 10400, Thailand; piyadac.fsn@afrims.org; 5 Institut des Sciences de l’Evolution—Montpellier University—National Centre for Scientific Research (CNRS)—French Agricultural Research Centre for International Development (CIRAD), 34090 Montpellier, France; 6 Faculty of Veterinary Technology, Kasetsart Univesity, Bangkok 10900, Thailand

**Keywords:** Trombiculidae, *Orientia tsutsugamushi*, identification characteristics, distance learning, educational video

## Abstract

Scrub typhus, a disease caused by *Orientia tsutsugamushi*, affects more than one billion people globally with an average fatality rate of 6%. Humans are accidentally infected through the bite of trombiculid mite larvae (chiggers). Chiggers feed on hosts’ extracellular fluid for survival and development. *O. tsutsugamushi* is maintained throughout the chigger’s lifespan and over several generations. Although disease-related knowledge is essential in designing effective control strategies, many personnel in related sectors are unfamiliar with this disease and its vector. To tackle this issue, we developed a distance learning tool using educational videos on scrub typhus- and vector-related topics. The learning method is facilitated online, and students and tutors are not required to be physically present at the same place and time, thus allowing flexibility and accessibility. Knowledge improvement of 34 participants from related sectors was evaluated by pre- and post-test questionnaires. Although 54% of participants had prior knowledge of scrub typhus, 76.5% still lack basic knowledge of vector identification. After the distance learning, the average score increased significantly from the baseline (*p* < 0.05). Most participants showed interest in the topic and learning method. These results suggest that the distance learning method was promising in distributing health-related information and might be applied to other diseases and communities.

## 1. Introduction

Scrub typhus is a neglected tropical infectious disease caused by *Orientia tsutsugamushi*, Gram-negative bacteria of the Rickettsia group [1]. It affects more than one billion people globally, with an average fatality rate of 6%. In Thailand, scrub typhus has been reported country-wide, with the highest endemicity in the northern and northeastern provinces [2]. During 2015–2019, the morbidity rate ranged from 10.9 to 14.7 per 100,000 population, with an average case fatality rate of 0.08 per 100,000 in the past 5 years [2]. Scrub typhus remains an important disease burden in Thailand, especially in populations with limited access to health education and treatment [3,4].

Scrub typhus is transmitted by the larval stage of trombiculid mites, often referred to as chiggers. Chigger is the only parasitic stage in the mite life cycle that can transmit the pathogen to the host. After hatching from eggs, chiggers emerge from the soil and seek hosts. When suitable vertebrate hosts pass their habitats, they use their claws to attach themselves to the hosts’ bodies. In contrast to hematophagous arthropods, chiggers feed on hosts’ extracellular fluid and liquefied tissue lysate, which they use for survival and completion of their development. To be fully engorged, chiggers have to feed on one or, in exceptional cases, two or more hosts. Afterward, they drop down to the soil and develop into three nymphal and adult stages [5,6,7,8]. The pathogen is maintained throughout the chigger’s lifespan and transmitted vertically to the successive generations.

Vertebrates, mainly rodents, serve as maintenance hosts for the chigger mite. Humans become accidentally infected when visiting the habitat of chigger mites and common reservoir hosts [6,7,8,9]. These high-risk areas include rice fields, forests, gardens, plantations, and various eco-tourism areas, such as national parks [10,11].

Knowledge of scrub typhus and its vector is essential for self-awareness and disease prevention. Thus, people working in related sectors, including public health officers, health care providers, eco-tourism staff, and researchers, are expected to possess basic knowledge of this problem. Unfortunately, despite the continuing scrub typhus epidemic, this disease did not receive high priority in Thailand. On the other hand, organizing workshops, seminars, or courses will require a high budget and special timing.

With the current technological advancement, space and time differences are no longer obstacles for interaction. The wide coverage of the internet and social media allows information to be transferred 24 h a day from almost anywhere in the world. In Thailand, 51 million people (approximately 74% of the total population) are listed as active social media users [12], mostly in the age range of 18–44 years old [13]. The most common social media used is Facebook, followed by YouTube. Internet traffic increased from 59% in 2014 to 77% in 2019. Furthermore, YouTube also reported that 300 h of video are uploaded every minute. The increasing video stream also reflects in the educational environment [14]. Thus, it is not surprising that the field of education also advances from this fact, one of which as distance learning [15].

Benefiting from the widespread technology and social media, distance learning has become an important element of education. This learning method engages people (tutors and students) who are physically distant from each other in a learning community sharing the same educational goals [16]. The study materials can be accessed anywhere and anytime inexpensively, thus providing convenience and flexibility to their users. Previous studies described that distance or online courses could be as effective as face-to-face courses [17,18]. One of the tools used in distance learning is video modules. Despite having been used for decades, not many studies have addressed the efficiency of video modules in delivering information to students [16].

In the present study, we aimed to develop a distance learning tool to better understand basic knowledge of scrub typhus and its vector. We also set out to evaluate the efficacy and perception of participants about the distance learning method. This work is of significant importance since an effective knowledge distribution will contribute to self-awareness, leading to the reduction of scrub typhus cases.

## 2. Materials and Methods

### 2.1. Study Participants

Volunteers were recruited to participate in this preliminary study. Investigators contacted each individual directly. We included males and females between 20 and 60 years old who were able to access the online videos and evaluation forms and came from healthcare sectors related to scrub typhus and/or chigger mites. The exclusion criteria comprised those who failed to complete the post-test questionnaire. Before enrolling in the study, each participant provided their consent. To protect the privacy of study participants, questionnaires were kept anonymous and results were reported as collective outcomes. This study had been approved by the Institutional Review Board of Department of Disease Control (ethical clearance approval number 63021).

### 2.2. Video Module

An educational video was developed based on outcome-based learning methods (Supplementary data). The expected outcome was basic understanding of scrub typhus (causative agent, the hallmark of symptoms and clinical characteristics, and transmission route), chigger mite as a scrub typhus vector (life cycle, habitat, risk area, and feeding behavior), and chigger mite morphological identification (main organ characteristics for identification). The video contained animated graphics, texts, and narration related to the topics. The study participants were requested to watch the educational video before finally filling in the post-test questionnaire.

### 2.3. Evaluation and Assessment of Participants’ Engagement

A structured questionnaire was developed both in English and in Thai. The questionnaire was pre-tested among participants with similar backgrounds to the population to be studied. Questionnaires were prepared using the Google form. To protect the privacy of study participants, questionnaires were kept anonymous. The participants were first asked to complete the pre-test questionnaire through a link to the corresponding Google form within a given time. The questionnaire was composed of three parts: demographic data of study participants, basic knowledge assessment of scrub typhus, and basic apprehension on chigger mite morphological identification. After completing the pre-test, the participants were given links or QR codes of the video modules. Finally, they were given a Google form link for the post-test questionnaire. The knowledge scores were calculated from the correct answers and compared between before and after the distance learning method.

After the distance learning, participants were also requested to answer additional questions regarding feedback on the distance learning method and its evaluation. The participants’ perception regarding the video and distance learning method was assessed using a Likert-type scale questionnaire, as described previously by Bayram [19]. It consisted of 17 (15 positive and 2 negative) items covering 4 sections: quality of contents, participant motivation, content comprehension, and technical aspects (Supplementary data). Using a 5-point scoring system, ranging from 1 as strongly disagree to 5 as strongly agree, the negative items were analyzed inversely.

### 2.4. Statistical Analysis

The results of pre- and post-test were presented as proportions. Chi-square was used to compare the categorical variables. The difference between proportions of pre- and post-test was assessed using the Z score. The mean scores of pre- and post-test were compared using Student *t*-test. Significance is considered when the *p*-value was less than 0.05.

## 3. Results

### 3.1. Demographic Data

We recruited 34 participants from related sectors, including graduate students, laboratory technicians, researchers, lecturers, public health officers, and health care providers. There were proportional numbers between male and female participants, with a median age of 31 years old. Most participants either had a Bachelor (32.4%), Master (32.4%), or Doctoral (26.5%) degree in related fields. Almost half of the study participants had prior training or experience on scrub typhus and ectoparasites (41.2% and 47.1%, respectively). The demographic data of participants are presented in Table 1.

### 3.2. Knowledge Improvement after the Distance Learning Method

Before the distance learning, participants’ background knowledge on this topic was evaluated by the pre-test questionnaire. Since almost half of them had prior experience or training on scrub typhus and ectoparasites, the average baseline score was high (72.1%). The lowest proportion of correct answers was found on the vector feeding habit and morphological identification topics (44.1% and 23.5%, respectively).

**Figure 1:**
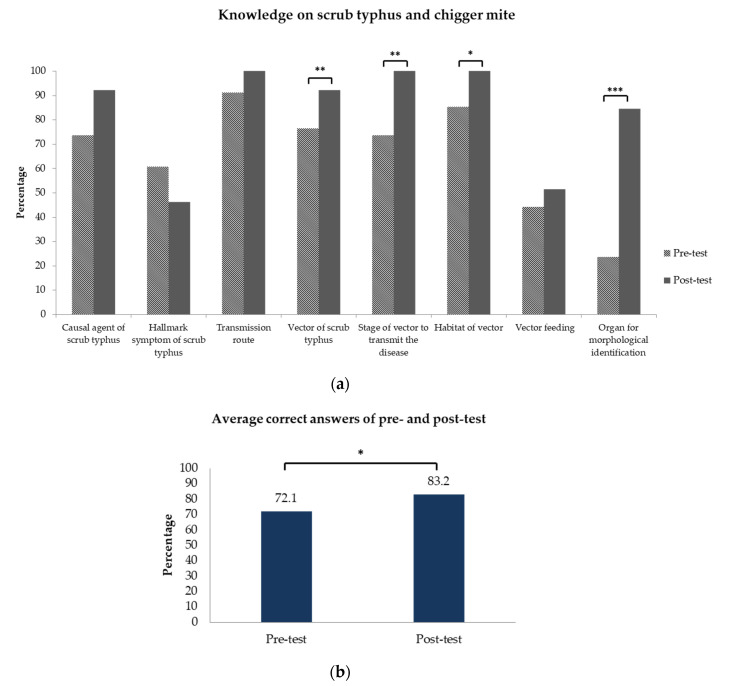
Knowledge improvement after the distance learning method: (**a**) the proportion of correct answers increased significantly on most questions; (**b**) the overall average of correct answers increased significantly after the distance learning method. * significant at *p* < 0.05; ** significant at *p* < 0.01; *** significant at *p* < 0.001.

After watching the video, the average score increased significantly to 83.2% (*p* < 0.05) (Figure 1b). The questions with significant improvement between pre- and post-test were vector of scrub typhus (76.5% vs. 92.3%, *p* < 0.01), stage of vector to transmit the disease (73.5% vs. 100%, *p* < 0.01), vector habitat (85.3% vs. 100%, *p* < 0.05), and most profoundly, organ for morphological identification (23.5% vs. 84.6%, *p* < 0.001), as shown in Table 2 and Figure 1a.

### 3.3. Morphological Identification of Chigger Mite

**Figure 2:**
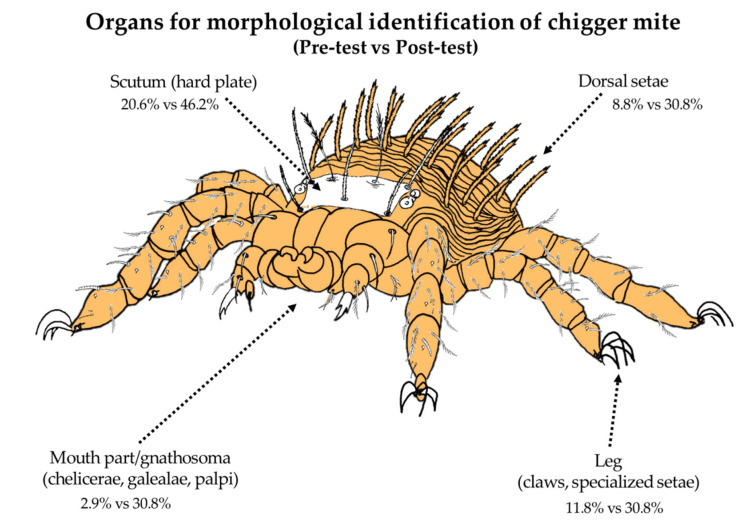
Morphological identification of chigger mites. Percentage shows correct answers of participants before versus after the distance learning method. Image courtesy of Rawadee Kumlert, Ph.D.

The participants were given an open-ended question about specific structures used in morphological identification of chigger mites. Common structures used in chigger mite identification include scutum (hard dorsal plate), dorsal setae, mouthpart or gnathosoma (chelicerae, galealae, and palpi), and legs (specialized setae and claws). Results from the pre-test revealed that only 23.5% of participants answered this part correctly, by giving at least one correct answer. After watching the video, this proportion increased significantly to 84.6%, *p* < 0.001 (Table 2). Most participants were able to recall and identify the organs used in chigger mite morphological identification (Figure 2).

### 3.4. Participants’ Feedback towards the Distance Learning Method

**Figure 3:**
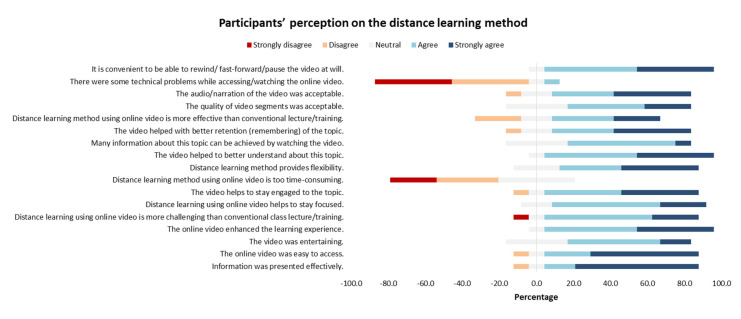
Participants’ perception on the distance learning method as evaluated by the Likert-type scale questionnaire. Colours indicate the level of agreement (strongly agree, agree, neutral, disagree or strongly disagree). Horizontal axis depicts percentage of participants’ agreement for each statement.

Participants’ engagement and feedback were assessed using a Likert-type scale questionnaire (Figure 3). Consisting of 17 (15 positive and 2 negative) items, it covered four sections. The first section assessed the quality of contents, which contained statements regarding effective presentation, video accessibility, entertainment, enhanced learning experience, and the challenging nature of this method. Overall, the participants showed agreement with the given statements, indicated by an average score of 4.15 ± 0.23 (mean ± SD). The second section about participants’ motivation included statements on participants’ focus and engagement, time consumption, and flexibility. The average response was 4.07 ± 0.15, showing the participants’ agreement. The third section on content comprehension mentioned the video’s role in better topic understanding, adequate information retention, and efficacy. This yielded an average of 3.93 ± 0.32, pointing out the neutral-to-agree response of participants. The fourth section discussed technical aspects of the video, including the segment quality, audio/narration, technical problems, and convenience. This final section had an overall mean of 4.4 ± 0.14, suggesting the participants’ agreement.

## 4. Discussion

We developed a distance learning method on scrub typhus and its vector using video modules. In this pilot study, the participants were recruited from related sectors, and almost half of them had prior training or experience in scrub typhus and ectoparasites, thus explaining the high baseline score of pre-tests. However, several topics, including scrub typhus vector, habitat, stage of vector, and key organs for morphological identification, were still less familiar. After engaging in our video, the participants’ knowledge of these topics increased significantly. Our result is in accordance with a study by Candarli, who utilized video conferences in higher education. Most of the students in their study stated that they learned important topics and felt that video conferencing gave positive benefits to education [16]. Previous studies described that distance or online courses could be as effective as face-to-face courses [17,20]. In Thailand, a study by Buakanok and colleagues described the use of video as educational media to improve health awareness. Similar to us, they also found respondents’ awareness of health issues to be significantly increased after watching the video [21].

The lowest proportion of correct answers was found on the vector morphological identification. Due to the very small size of chiggers (less than 1 mm), they cannot be observed by the naked eye [22]. To identify chiggers, experienced microscopic technique and permanent sample-preparation techniques are required. Recently, there are almost 3000 identified species of chigger worldwide [23], which are classified by their morphological characteristics of the dorsal and ventral part. However, some species in the same genus show similar characteristics. Thus, experience and understanding of basic morphological characters are important for those interested in studying in this field. Species identification of the medically important vector [23] and the biological, ecological, and epidemiological significance [24] of the disease are a necessity to study scrub typhus.

After watching the video modules, participants’ knowledge of basic chigger morphological identification increased significantly from 23.5% to 84.6% (*p* < 0.001). This indicated that online videos might be considered as a useful tool for training and improving participants’ knowledge. A study by Stefanidis and colleagues used video tutorials in the training of laparoscopic suturing skills. The participant feedback showed that the video provided a good proficiency level, required short training time and number of repetitions, and reduced the training costs [25].

The Likert-type scale questionnaire was used to assess participants’ engagement and feedback on the quality of contents, participants’ motivation, content comprehension, and technical aspects. The overall mean of each part indicated participants’ agreement with this method. The highest agreement was toward the effective presentation, accessibility, engagement, flexibility, ease in understanding, narration, and convenience to control the video at will. Our results are in accordance with the study by Manowong (2016), which involved facilitating students’ learning experiences using a social networking site called Edmodo. Their Likert-type survey revealed that Edmodo was perceived as a useful and beneficial learning tool since it provided various features to support the learning process. It was considered an effective learning tool as it increased students’ motivation, flexibility, and participation in online learning activities [26].

A major advantage perceived by 41.7% of participants was that the distance learning method provided flexibility. A study by He using online video tutorials in an analytical chemistry course also mentioned that they were valuable, flexible in time and place, and cost-effective [27]. Another study using a mobile live video learning system also mentioned that this method was practical and cost-efficient [28].

Using the Likert-type questionnaire, participants were given a chance to self-reflect and provide feedback on the distance learning method. Most participants felt engaged (83.4%), focused (83.3%), well-informed (66.6%), and better understood (91.7%) about the topics. This was in agreement with previous studies, which mentioned that videos could serve as a powerful teaching tool and provide key values of the educational context, including cognitive, experiential, and nurturing values [29]. Video-based learning also seemed to provide higher learner satisfaction, and video can be used as a reflection tool [30].

## 5. Conclusions

Overall, these preliminary results suggest that the distance learning method is promising in distributing health-related information. In the future, we plan to improve and test this method in more heterogeneous communities. Adequate disease-related knowledge in the community will contribute to more effective prevention strategies to reduce the disease burden.

## Figures and Tables

**Table 1:** Demographic data of study participants.

-	Variables	Number	(%)
Gender	Male	19	(55.9)
	Female	15	(44.1)
Age	20 to 29	15	(44.1)
	30 to 39	17	(50.0)
	40 to 49	2	(5.9)
Nationality	Thai	30	(88.2)
	Non-Thai	4	(11.8)
Education	High school	2	(5.9)
	Bachelor degree	11	(32.4)
	Master degree	11	(32.4)
	Doctoral degree	9	(26.5)
	Others	1	(2.9)
Occupation	Student	6	(17.6)
	Laboratory technician	6	(17.6)
	Researcher	6	(17.6)
	Lecturer	3	(8.8)
	Public health officer	7	(20.6)
	Others	6	(17.6)
Prior experience/training on scrub typhus	Yes	14	(41.2)
	No	19	(55.9)
Prior experience/training on ectoparasites	Yes	16	(47.1)
	No	18	(52.9)

**Table 2:** Knowledge of study participants on scrub typhus and chigger mite.

**Variables**	**Pre-Test**	**(%)**	**Post-Test**	**(%)**	**Chi-Square**	**Z-Score**
Causal agent of scrub typhus	25	(73.5)	31	(92.3)	2.52	1.91
Hallmark symptom of scrub typhus	21	(60.6)	16	(46.2)	0.95	1.22
Transmission route	31	(91.2)	34	(100)	NA	1.77
Vector of scrub typhus	26	(76.5)	31	(92.3)	1.74	** 3.01
Stage of vector to transmit the disease	25	(73.5)	34	(100)	NA	** 3.22
Habitat of vector	29	(85.3)	34	(100)	NA	* 2.32
Vector feeding	15	(44.1)	18	(51.5)	0.24	0.73
**Vector Morphological Identification**	**Pre-Test**	**(%)**	**Post-Test**	**(%)**	**Chi-Square**	**Z-Score**
Organs for morphological identification	8	(23.5)	29	(84.6)	*** 23.71	*** 5.11
Scutum	7	(20.6)	16	(46.2)	-	-
Setae	3	(8.8)	10	(30.8)	-	-
Legs	4	(11.8)	10	(30.8)	-	-
Mouth part	1	(2.9)	10	(30.8)	-	-
Others	0	(0.0)	5	(15.4)	-	-

* significant at *p* < 0.05; ** significant at *p* < 0.01; *** significant at *p* < 0.001.

## Supplementary Materials

The following are available online at https://www.mdpi.com/2414-6366/5/2/55/s1, Video S1: Basics of scrub typhus and chigger mite vector https://www.youtube.com/watch?v=1eX2MIB5_no&t=24s, Video S2: Chigger mite’s life cycle and feeding site https://www.youtube.com/watch?v=sAe14qm7ZjM, Video S3: Chigger collection and basic identification https://www.youtube.com/watch?v=DHzEg1vf-1I, pre-test questionnaires: https://forms.gle/iFuaRm7khJRPiRD78, post-test questionnaires: https://forms.gle/oC9iZezVvN7rqC779 and Likert Scale on perception of the online video used in the distance learning method: https://forms.gle/sFDRXDRKLuVpmJyX7.
